# *Spodoptera frugiperda* Smith (Lepidoptera: Noctuidae) in Cameroon: Case study on its distribution, damage, pesticide use, genetic differentiation and host plants

**DOI:** 10.1371/journal.pone.0215749

**Published:** 2019-04-29

**Authors:** Apollin Fotso Kuate, Rachid Hanna, Armand R. P. Doumtsop Fotio, Albert Fomumbod Abang, Samuel Nanga Nanga, Sergine Ngatat, Maurice Tindo, Cargele Masso, Rose Ndemah, Christopher Suh, Komi Kouma Mokpokpo Fiaboe

**Affiliations:** 1 International Institute of Tropical Agriculture (IITA), Yaoundé, Cameroon; 2 University of Maroua, Faculty of Science, Maroua, Cameroon; 3 University of Douala, Faculty of Science, Douala, Cameroon; 4 Institute of Agricultural Research for Development (IRAD), Yaoundé, Cameroon; University of Tennessee, UNITED STATES

## Abstract

Maize farmers in sub-Saharan Africa recently experienced unusual damage in their farms, attributed to the fall armyworm (FAW) *Spodoptera frugiperda* (J. E. Smith). This pest was first recorded in Africa in 2016, but detailed information on its distribution and damage and farmer’s response in invaded areas are largely lacking. In this study, we determined FAW distribution, genetic diversity, host plants, crop damage, and farmers’ responses. *S*. *frugiperda* was recorded in the 10 regions of Cameroon. Average percentage of infested plants and damage severity (on a scale of 1 to 5) were lowest—20.7 ± 7.4% and 2.1 ± 0.1 respectively—in the Sahelian regions and greatest—69.0 ± 4.3% and 3.1 ± 0.1 respectively—in the Western Highlands. Altitude did not influence FAW incidence and severity and its larvae infrequently co-occurred with maize stemborers on the same plants, suggesting possible direct and/or indirect competition between the two groups of maize pests. In response to this new threat to maize production, farmers have opted for the application of synthetic pesticides. Although our experiments were not designed to determine pesticide efficacy, as parameters such as time since application were not considered, our observations suggest lack of a drastic effect on *S*. *frugiperda* infestations on maize. There were two haplotypes of FAW co-occurring in Cameroon corresponding to the rice and corn strains and separated by 1.7% sequence divergence, which does not support the existence of cryptic species. *S*. *frugiperda* larvae were also recorded on *Sorghum bicolor* (L.) Moench (10.6%), *Solanum tuberosum* L. (2.8%), *Ipomoea batatas* (L.) Lam. (1.9%), *Saccharum officinarum* L (0.8%), *Phaseolus vulgaris* L. (0.4%) and *Gossypium hirsutum* L. (1.9%). This study show that two strains are present in all agroecological zones in Cameroon, and probably in neighboring countries of central Africa sharing the same agroecologies. Management options should therefore consider the use of specific natural enemies and an informed decision of intervention based on strain capture and damage threshold, to avoid pesticide resistance that may arise from inadequate use of chemicals. Further studies should also be undertaken to assess the response of the two *S*. *frugiperda* strains to biopesticides and botanical insecticides.

## Introduction

Maize (*Zea mays* L.) remains one of the most important crops in tropical areas and constitutes with wheat and rice, the main proportion of daily food intake of the inhabitants [1–3]. World maize production was estimated at 1,291 million tons in 2016 [4].

In Cameroon, maize provides almost half of the calories consumed in both rural and urban areas. It is largely grown in the west and northwest region of the country and has increasingly become a staple food in many parts of the country. Largest share of maize production is attributed to small-scale farmers and constitute a direct source for household livelihoods [5].

Maize production in Cameroon like in many other crops in sub-Saharan Africa are hampered by many biotic and abiotic factors, including poor soils, droughts, crop pests, diseases and weeds, and unsuitable temperatures [6–10]. A classification of the most important maize pests was given by [11] who identified several species of moths as the most damaging to maize worldwide. The group includes cutworms, armyworms, earworms, borers and grain moths and followed by the beetles (rootworms, wireworms, grubs, grain borers, and weevils). Other important groups of insects—thrips and sap-sucking bugs (leafhoppers and aphids) [11]—serve as vectors for disease agents (viruses, mycoplasms, bacteria, and fungi).

*Busseola fusca* Fuller (Lepidoptera: Noctuidae) has been considered for long the most destructive lepidopteran pest of maize and sorghum in Africa [12–14]. Despite the huge amount of information about its management, this pest still represents a major constraint to maize production in areas where it is abundant. Yield losses due to *B*. *fusca* damage on maize varies between 14% in Kenya and 40% in monocropped maize fields in Cameroon [15,16].

The African armyworm (*Spodoptera exempta* Walker) is also known as a major pest of maize, sorghum, millet and rice, causing yield loss by defoliation [17]. Control during outbreaks are achieved through agricultural practices (planting time and timely weeding), biological control with *Spodoptera exempta* nucleopolyhedrovirus (SpexNPV) and chemical application with a wide range of pesticides, excluding all the chlorinated hydrocarbons such as DDT and BHC [17]. In addition to the damage caused by this native lepidopteran species on maize, farmers in sub-Saharan Africa recently experienced unusual damages in their farms caused by *Spodoptera frugiperda* (J. E. Smith). *S*. *frugiperda* is a Lepidopteran species of the Noctuidae family, whose caterpillars are crop pests. The caterpillar, which feeds on several plants, is known for its damage on various crops, including maize (*Zea mays* L.), sorghum (*Sorghum bicolor* (L.) Moench) and cotton (*Gossypium* sp. L.) [18].

Native to the Americas, *S*. *frugiperda* was first detected in Africa in 2016 [19]. It has been officially confirmed in over 44 countries in sub Saharan Africa [18]. Infestations by *S*. *frugiperda* during the maize stages may result in yield losses of 15 to 73% when 55 to 100% of the plants are infested [20]. The larvae appear to be much more damaging to maize in West and Central Africa than most other African *Spodoptera* species [21].

Maize yield loss extrapolated from 12 African countries, based on studies in Ghana and Zambia, is estimated between 8.3–20.5 mil tons, worth between US$2.5–6.2 billion [22]. Interventions based on pest incidence thresholds are primarily intended to better protect seedlings and reproductive stages of maize. However, early detection is essential, as the application of chemical insecticides is effective only on young larval stages [18].

The presence of *S*. *frugiperda* was detected in maize farms in the west region of Cameroon, in December 2015 [23]. However, no quantitative data, nor the extent of its damage or its distribution is available for central Africa. Initial yield losses due to *S*. *frugiperda* in Cameroon is estimated between 0.3–0.8 mil tons, worth between US$ 0.1–0.8 billion [22], with a 25 billion sector at risk per year, although these estimates are extrapolations from other areas in Africa. As a response to *S*. *frugiperda* invasion in Cameroon, a network of stakeholders, including government and research institutions, has developed a national strategic plan to address the *S*. *frugiperda* threat to Cameroon agriculture production. The information presented here consists of baseline studies, in the framework of the Cameroonian national plan, to identify *S*. *frugiperda* populations, improve understanding of their distribution, damage, and host plants, evaluate the perception of farmers and identify potential natural enemies. This information will be useful in designing an integrated control approach and could serve as example for other countries sharing similar agroecologies with Cameroon in Central Africa.

## Materials and methods

### Study sites

**Fig 1:**
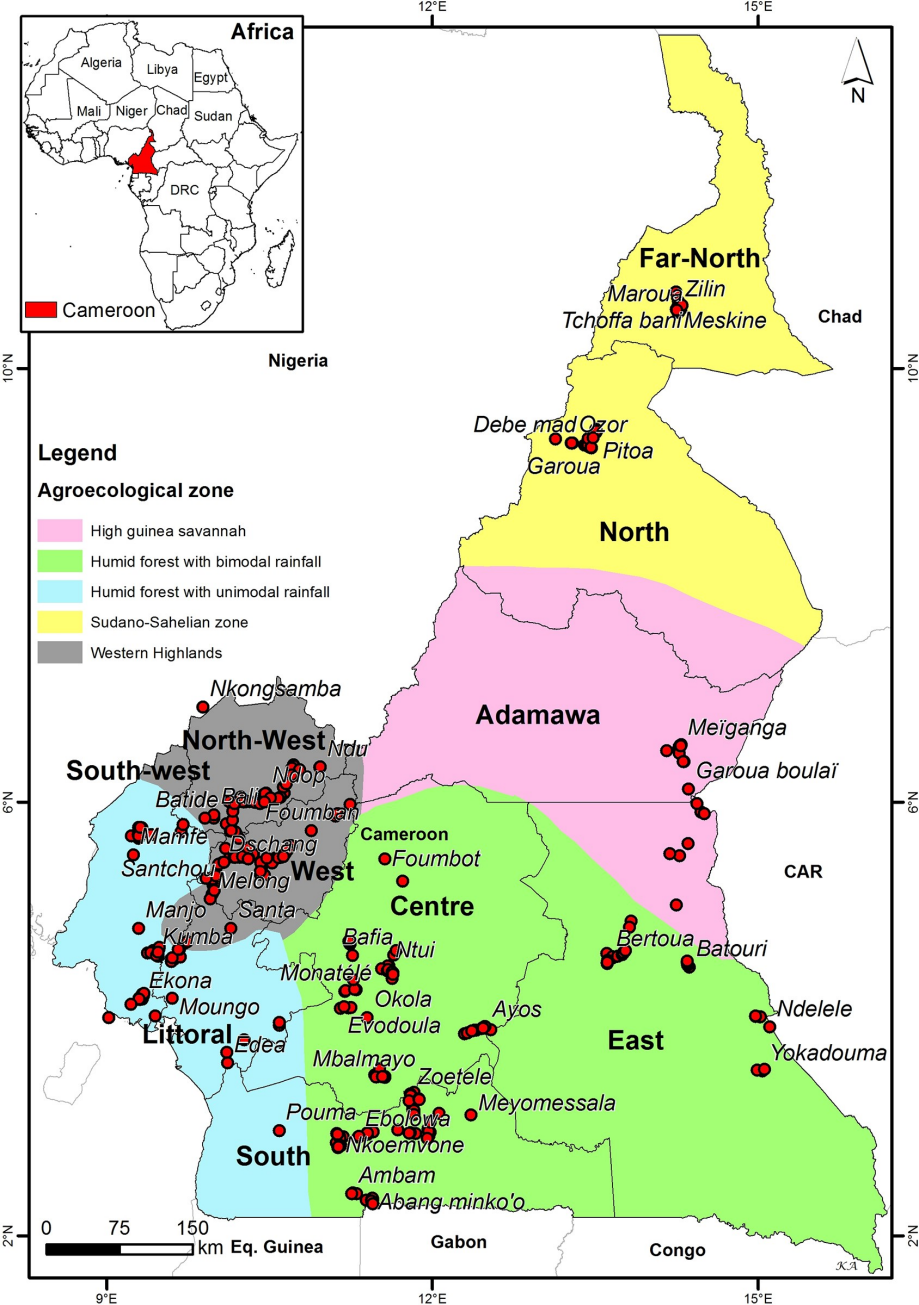
Sampling locations during the three rounds of surveys in the five agroecological zones of Cameroon.

Three rounds of survey were conducted between February and March, May and June, October and November 2017 respectively in the ten regions of Cameroon (Fig 1). The regions are grouped into five agroecological zones namely the Soudano-sahelian zone (North and Far North regions), the High Guinea savannah (Adamawa region), the humid forest with bimodal rainfall (Center, South and East regions), the humid forest with Monomodal rainfall (Littoral and Southwest regions) and the western Highlands (West and Northwest regions). The first survey was done during the dry season during which maize is only grown in swamps. The two other survey periods corresponded to the usual maize cropping periods during the rainy season in the respective areas. The study was carried out on private land and the owner of the land gave permission to conduct the study in his farm. No specific permissions were required to conduct activities in these locations as per IITA host country agreement with the government of Cameroon. The field studies did not involve endangered or protected species as we worked in farmer’s maize farms. A total of 420 fields were surveyed in 261 villages grouped in 61 locations. The number of fields sampled in each location was based on availability of maize fields at the time of sampling (Table 1).

**Table 1:** Number of fields sampled during the three surveys.

Regions	Survey #1	Survey #2	Survey #3	Total
Adamawa	4	5	2	11
Center, East and South	53	52	68	173
North and Far North	-	13	51	64
Littoral and Southwest	19	20	26	65
West and Northwest	31	35	41	107
Total	107	125	188	420

### Sampling methods and data collection

In each region, one field was sampled per village. Two consecutive fields were separated by at least 20 km, unless constrained by the availability of fields. In each field, geographic coordinates (using hand held Garmin GPS) and other field information such as field size, surrounding and within field vegetation were recorded. The owner of the field was also interviewed using a short, structured questionnaire to obtain information about farm management practices such as planting date, variety, source of seeds, pesticides type and frequency, fertilizer type, type of recent change in infestation, first experience of damage increase. Participating farmers gave their oral consent before we proceeded, and the collected data was treated anonymously.

Each sample field was divided into four equal plots. In each plot, scouting was done by inspecting 10 plants, moving along a W-shape design. The middle of the field was also sampled, making 50 plants surveyed per field. Distance between two consecutive plants was a function of field size and shape but was representative of the plot area [18].

*Spodoptera frugiperda* presence was determined using the following indicators: (i) presence of fresh frass in the leaf funnel; (ii) presence of larvae on leaves or in the leaf funnel identifiable with the inverted Y-Shape in the head and the set of four dot forming a square on the upper surface of the last segment of its body [18]; (iii) irregular damage (cuts) on leaves and (iv) presence of egg masses. Other plants known to be *S*. *frugiperda* hosts were also inspected [22,24].

The co-occurrence of maize stemborer larvae (e.g., *Busseola fusca* Fuller) and *S*. *frugiperda* on the same plants was also recorded to evaluate possible competitive interactions between the two pests.

Field sampling was destructive; the inspected plants were cut from the ground level and dissected to record the number of *S*. *frugiperda*, stemborers, egg batches and any other insects present. Damage to the plant was also scored by evaluating severity of pin holes, shot-holes, lesions, tattering and dead hearts. A rating scale from 1 to 5 was used for scoring of damage severity on whorl-stage plants as follows:

1. Healthy maize without damage;
2. 1–10% leaf damage or presence of damage from fall armyworm limited to characteristic windows or < 5 mm diameter and or destruction of only the leaf cuticle;
3. 11–25% leaf damage with presence of chewed areas > 5 mm, funnel leaves still intact;
4. 26–50% leaf damage with presence chewed areas larger than 1 cm, the funnel slightly damaged or less severe;
5. > 50% leaf damage, plant stunting and funnel damaged severely [25,26].

All larvae for genetic studies were kept in 90%-alcohol and brought to the laboratory. Another batch of larvae was used to establish FAW colonies in the laboratory.

### Molecular characterization of *S*. *frugiperda* populations

#### DNA extraction, amplification and sequencing

To determine the genetic diversity of *S*. *frugiperda* populations in Cameroon, DNA sequence data for mitochondrial Cytochrome c oxidase subunit 1 (COI), encoding gene commonly used for ‘DNA barcode’ [27], were obtained from 95 specimens collected from 27 locations across the country during the surveys and kept in 95% ethanol. The collection included 71 specimens of the fall armyworm and 24 specimens of other stemborers. All specimens examined were larvae collected from maize. DNA extraction and amplification was done at the molecular laboratory facilities of the International Institute of Tropical Agriculture (IITA, Yaoundé Nkolbisson, Cameroon). A fragment of each specimen was cut from the caudal end of the worm body and put in individual Eppendorf tubes. DNA was extracted following the manufacturer’s insect specific protocol for the DNEasy Blood and Tissue kit (P/N 69506; QIAGEN, GmbH) [28–30]. After extraction, DNA was stored at ‐20°C for pending use. Two microliters (2 μl) of the extracted DNA were used as a template in 25 μl final volume of PCR reactions. The primer pairs LepF (5_-ATTCAACCAATCATAAAGATATTGG-3_) and LepR (5_-TAAACTTCTGGATGTCCAAAAAATCA-3_) were used to amplify an approximately 700 bp DNA fragment of mitochondrial COI. The thermocycling profile consisted of: initial denaturation at 94°C for 1.5 min, followed by 35 cycles of (denaturing at 95°C for 30s, annealing at 55°c for 1 min and elongation at 72°C for 1.5 min), final elongation took place at 72°C for 7min and a final lap at 4°C [31]. Five microliters (5 μl) of the amplified DNA were visualized on 2% agarose gel and 20 μl of the amplified product was shipped to Inqaba Biotec South Africa for sequencing. Amplified DNA was subsequently purified and sequenced in both directions using the same primers, yielding a 658-base pair “barcode”.

### Data analysis

*Spodoptera frugiperda* incidence and severity values were obtained for each of the surveyed maize fields. Incidence was calculated as the percentage of plants infested by S. *frugiperda* while damage score was calculated by dividing the sum of score (excluding score 1 –absence of *S*. *frugiperda*) by the number of plants damaged. We used the spearman correlation to evaluate the effect of altitude and field age, on *S*. *frugiperda* damage and severity. We used nested ANOVA to compare *S*. *frugiperda* incidence and severity between regions and seasons while considering location as random factor nested in regions, and regions and seasons as fixed factors. We used in the ANOVA log-transformed average incidence and severity per village to reduce heteroscedasticity inherent in our type of data [32].

#### Phylogenetic analyses

Raw sequences were trimmed in Chromas (version 2.6.2) by removing primer regions from both the forward and reverse strands. Forward and reverse sequences were assembled, and consensus sequences were created in BioEdit 7.0.9.0 [33]. All consensus sequences resulted in exactly 658 bp perfectly aligned. To further confirm the species identity, specimens were analyzed by ‘DNA barcoding’. The DNA sequence of each specimen was blast using BLASTn on the NCBI GenBank database and the data coverage and identity percentage were noted. To ensure sequences quality, DNA sequences were translated into proteins by assigning the invertebrate mitochondrial genetic code and setting a 3, 1, 2 codon position using Mesquite (version 3.5), which resulted in protein sequences with no gaps and no stop codons. The DNA sequence dataset was completed with some *S*. *frugiperda* COI sequences from previous studies [19,34,35], retrieved from the NCBI GenBank database. Multiple sequence alignments for the sequence data were then generated in MAFFT [36]. Pairwise genetic distances were assessed for the Cameroon populations of *S*. *frugiperda* and the dataset was subjected to neighbor joining (NJ), maximum parsimony (MP) and Maximum likelihood (ML) tests using MEGA 7.0.21. Prior to the tests, the best-fit substitution model was determined using the highest Akaike (AIC) and Bayesian (BIC) Information Criterion. Since sequences retrieved from GenBank were of shorter length, their alignment with sequences obtained in this study lead to the creation of gaps at the 3’ end. These gaps were treated in the analysis as missing data. Neighbor-Joining (NJ) analysis was carried based on p-distances and a pairwise deletion option for gaps treatment. For Maximum Parsimony (MP) analysis, phylogenetic trees were constructed using heuristic searches with a bootstrap method (1,000 replicates) and Subtree-Pruning-Regrafting (SPR) branch swapping. Phylogenetic reconstruction using maximum likelihood was performed with a bootstrap method (1,000 replicates) and a nearest-neighbor-interchange heuristic search method. *Spodoptera littoralis* Boisduval was used as outgroup in the subset of *S*. *frugiperda* populations while *Eldana saccharina* Walker was used as outgroup in the analysis of the whole dataset including other stemborers.

## Results

### Distribution and damage severity of the *S*. *frugiperda* in Cameroon

*S*. *frugiperda* presence was recorded in all 10 regions of Cameroon, with incidence ranging from 22.9 ± 5.7% in the Far North region recorded during the second survey, to 79.2 ± 3.4% in the West region during the third survey. Damage severity ranged from 2.15 ± 0.08% in the Far North region recorded during the second survey, to 3.64 ± 0.10% in the Littoral region during the second survey. Average larva count ranged from 6.33 ± 1.51 to 19.78 ± 3.03 in untreated plots while it was between 4.43 ± 1.73 to 29.0 ± 2.70 in treated plots. Greater number of larvae per field were recorded in the West region (20.1 ± 2.30) followed by the East (15.9 ± 2.45) and the Northwest (15.3 ± 2.14). The lowest number of larvae was recorded in the Far north region (6.59 ± 1.39) (Table 2).

**Table 2:** Mean ± SE of *S*. *frugiperda* incidence and severity in the various regions averaged between fields.

Region	Mean Incidence (% of infested plant)			Mean severity (on a scale of 1 to 5)			Average FAW larvae count		
	Survey #1	Survey #2	Survey #3	Survey #1	Survey #2	Survey #3	Untreated plot	Treated plot	Combined
Adamawa	41.5 ± 4.1	34.0 ± 16.5	60.0 ± 4.0	2.29 ± 0.10	3.27 ± 0.20	3.02 ± 0.12	9.36 ± 4.97	-	9.36 ± 4.97
Centre	71.4 ± 4.2	29.2 ± 6.2	54.4 ± 4.2	2.61 ± 0.09	2.91 ± 0.14	2.95 ± 0.08	11.1 ± 1.98	15.0 ± 3.18	12.1 ± 1.68
East	64.7 ± 8.4	37.6 ± 10.2	60.7 ± 5.4	2.46 ± 0.10	3.10 ± 0.22	3.13 ± 0.07	15.2 ± 2.33	29.0 ± 2.70	15.9 ± 2.45
Far North	NA	22.9 ± 5.7	37.4 ± 4.0	NA	2.15 ± 0.08	2.29 ± 0.04	7.20 ± 1.71	4.43 ± 1.73	6.59 ± 1.39
Littoral	40.4 ± 15.4	54.0 ± 10.4	69.7 ± 4.7	2.72 ± 0.21	3.64 ± 0.10	2.58 ± 0.09	10.9 ± 2.22	5.57 ± 3.88	9.18 ± 1.97
North	NA	50.7 ± 12.3	40.2 ± 4.3	NA	2.42 ± 0.13	2.34 ± 0.05	6.33 ± 1.51	7.00 ± 1.93	6.75 ± 1.31
Northwest	51.9 ± 4.8	44.7 ± 4.6	74.8 ± 3.7	2.96 ± 0.09	3.51 ± 0.13	2.83 ± 0.07	13.2 ± 2.45	19.7 ± 4.13	15.3 ± 2.14
South	73.4 ± 3.7	40.4 ± 4.8	55.2 ± 4.7	2.42 ± 0.06	3.00 ± 0.09	2.60 ± 0.04	11.7 ± 1.82	13.6 ± 2.92	12.1 ± 1.55
Southwest	58.7 ± 6.4	38.5 ± 3.8	75.4 ± 5.8	2.93 ± 0.12	3.28 ± 0.14	2.72 ± 0.08	12.7 ± 1.67	17.1 ± 2.65	14.9 ± 1.59
West	65.6 ± 4.6	54.0 ± 5.0	79.2 ± 3.4	3.23 ± 0.11	3.29 ± 0.12	2.76 ± 0.08	19.8 ± 3.03	20.6 ± 3.58	20.1 ± 2.3
Average	58.4 ± 4.5	40.6 ± 3.3	60.7 ± 4.6	2.70 ± 0.11	3.06 ± 0.14	2.72 ± 0.09	12.6 ± 0.08	14.5 ±1.25	13.2 ± 0.67

(NA = not available)

Mean incidence was significantly greater during the first and the third survey (58.4 ± 4.5% and 60.7 ± 4.6% respectively) compared with the second survey (40.6 ± 3.3%). However, mean severity during the second survey (3.06 ± 0.14) was greater compared with the first and the third survey (2.70 ± 0.11 and 2.72 ± 0.09 respectively) (Table 3).

**Table 3:** Mixed model analysis of *Spodoptera frugiperda* incidence and severity between regions, seasons and locations.

Y	Source	SS	MS Num	DF Num	F Ratio	Prob > F
Mean Severity	Survey	7.91	3.96	2	25.07	<0.0001
	Region[Location]	1.23	0.62	2	3.91	0.02
	Location&*Random*	27.07	0.45	60	2.86	<0.0001
Mean Incidence	Survey	30237.2	15118.6	2	36.11	<0.0001
	Region[Location]	606.70	303.35	2	0.72	0.49
	Location&*Random*	44571.4	742.86	60	1.77	<0.0001

There was a positive and significant correlation between *S*. *frugiperda* incidence and damage severity (r = 0.21, n = 416, P< 0.0001); and a negative correlation between incidence the *S*. *frugiperda* and the age of the plants (r = -0.13, n = 420, P < 0.01) (S1 Fig).

### Characteristics of the fields and pesticide use by farmers

Maize field size ranged from 0.02 to 1.7 ha (mean ± SE: 0.18 ± 0.02 ha) while maize plants age ranged from 12 and 88 days (mean ± SE: 58.5 ± 1.3 days). About 87% of the plant sampled were at the early whorl stage (VE-V6), while 13% was at the late whorl stage to silking (V7-R3).

**Fig 2:**
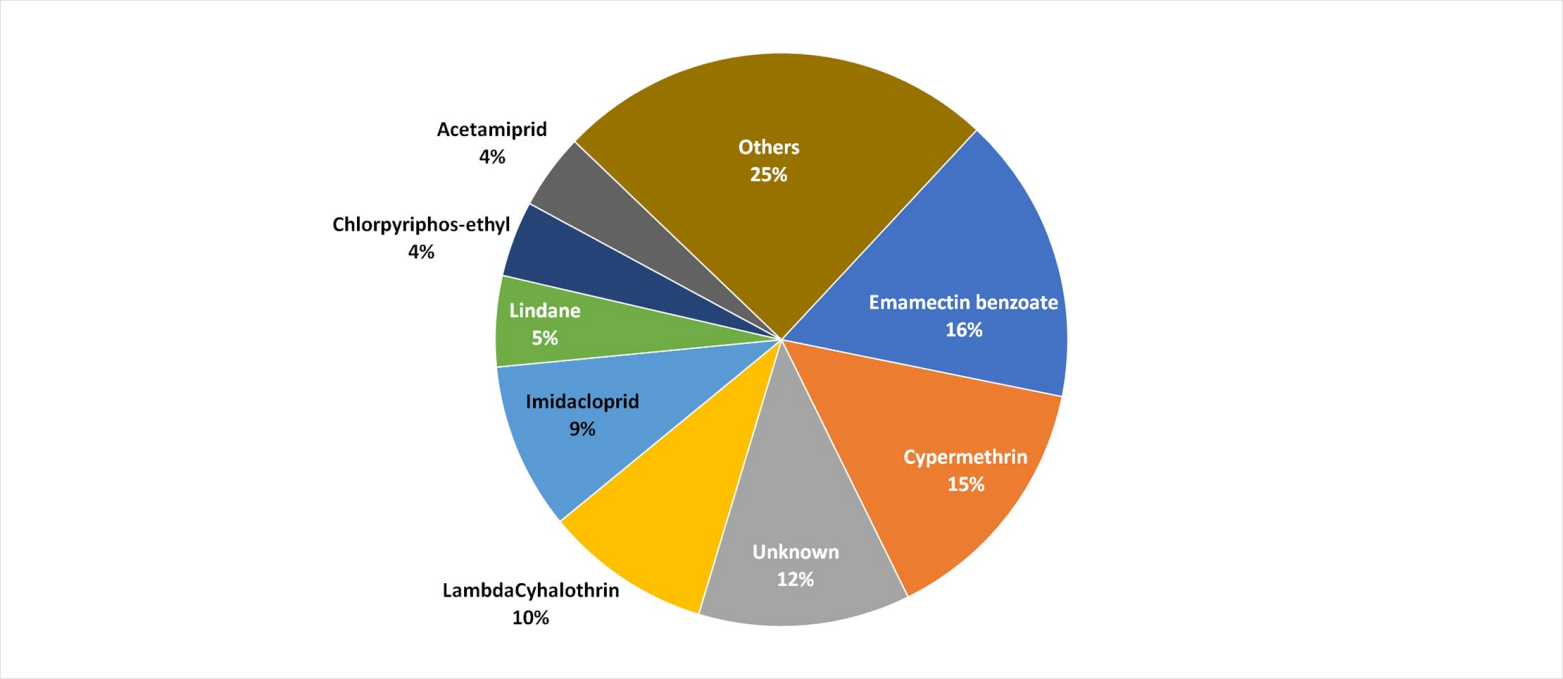
Active ingredients used by farmers to control *S*. *frugiperda* infestation on maize.

Twenty-six percent of the respondents treated their field with pesticides in response to *S*. *frugiperda* infestations. We recorded 3,706 larvae of all stages in untreated plots (n = 294 fields) and 1,832 larvae of all stages in treated plots (n = 126 fields). The difference between treated and untreated plots was not significant (F_(1, 418)_ = 1.95, P = 0.16). Emamectin benzoate was the most common active ingredient mentioned by farmers (16%), followed by Cypermethrin (15%) and Lamdacyhalothrin (10%). Only one farmer said to have used wood ash initially and a synthetic chemical afterwards. Twelve percent of the farmers did not recall the name of the pesticides used (Fig 2).

**Fig 3:**
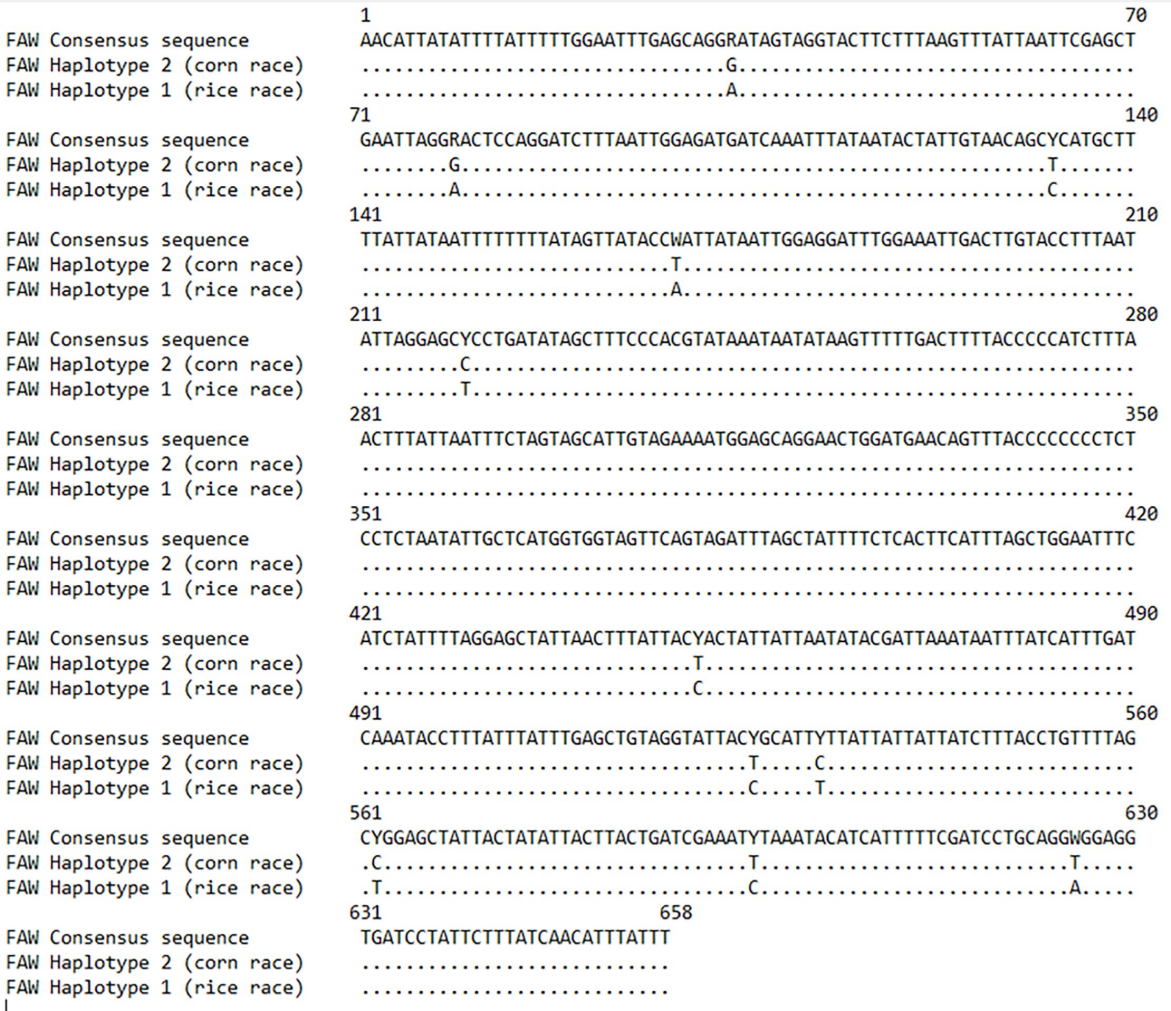
Pesticide application frequency to control *S*. *frugiperda* on maize by farmers.

Pesticides were applied at a frequency varying from twice a week (9%) to once during the crop cycle (15%) (Fig 3). Pesticide use did not reduce *S*. *frugiperda* damage incidence (treated fields: 55.9 ± 7.6%; untreated fields: 50.8 ± 4.3%, t = 1.98, P = 0.05) or severity (treated fields: 2.84 ± 0.08; untreated fields: 2.86 ± 0.08, t = 1.27, P = 0.20).

### Identification of *S*. *frugiperda* strains

The ninety five specimens used in this study were medium to large size larvae and were presented by S1_FAW—S95 FAW (S1 Table) and grouped into *S*. *frugiperda* samples (72 specimen) and stemborer samples as outer group (23 specimen). They were accessioned in the Laboratory of Entomology of IITA Cameroon. Based on the BLASTn analyses of the 658 bp COI sequence data on the NCBI GenBank database, the specimens were identified as *Spodoptera frugiperda* Smith (98.6%) and *Spodoptera litura* Fabricius (1.6%). This identification is supported by 98 to 100% similarity in sequence data and coverage (S1 Table).

### Genetic distance of *S*. *frugiperda* populations

**Fig 4:**
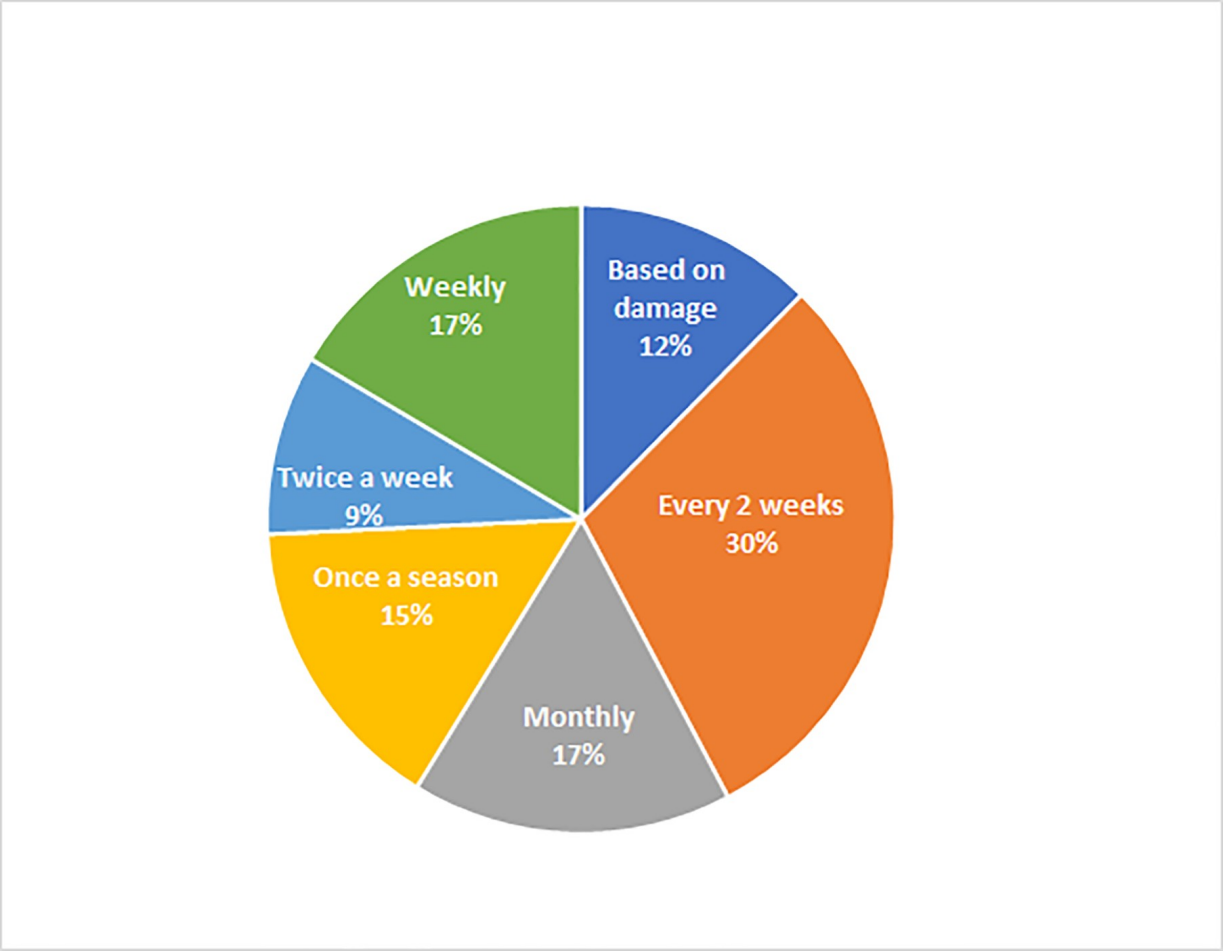
Identity plot of the COI sequences of *S*. *frugiperda* haplotypes from Cameroon.

Based on the genetic distances, *S*. *frugiperda* specimens fell into two discrete haplotype groups (see the phylogenetic tree). All sequences in each group are completely identical (null genetic distance) while the two groups differed by 1.7% sequence divergence resulting from 11 nucleotide base substitutions (Fig 4). However, there was not any change of amino acids in the resulting 219 amino acids protein sequence of the two haplotypes (S2 Fig).

### Phylogenetic relationship of *S*. *frugiperda* populations

**Fig 5:**
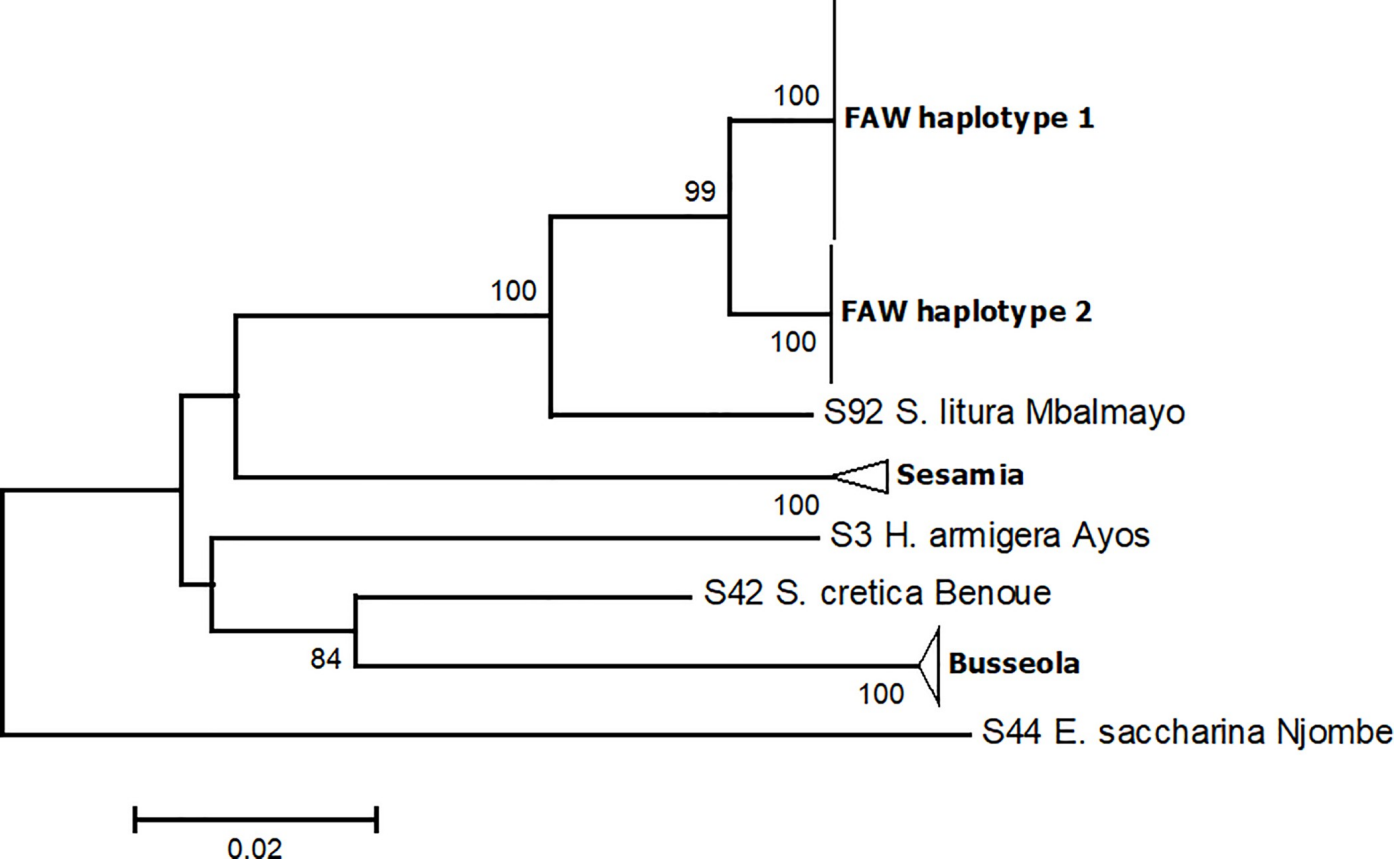
Phylogenetic relationship of 71 samples of the fall armyworm *Spodoptera frugiperda* populations from Cameroon, inferred from the 658 bp mitochondrial cytochrome c oxidase subunit 1 (COI) using Neighbor Joining method based on p-distances; scale unit is the number of base differences per site. The percentage of replicate trees in which the associated taxa clustered together in the bootstrap test (1000 replicates) are shown next to the branches. Evolutionary analyses were conducted in MEGA7.

The best-fit substitution model determined by the AIC and BIC was the Jukes-Cantor model. Fall armyworm collections from Cameroon resolved into two distinct clades with no sequence variation within clades (Fig 5). Forty-five specimens out of 71 grouped into the first clade (haplotype 1) including specimens from Brazil, Costa-Rica, Tanzania, Nigeria and especially the “rice” strain from the USA, while the 26 other specimens grouped into the second clade (haplotype 2) with specimens from Sao-Tome and especially the “corn” strain from the USA. The same clusters were well supported by MP and ML analyses (S3 and S4 Figs respectively). The relationship with other stemborers is also provided (S5 Fig).

### Distribution of haplotypes in surveyed agroecologies

Specimens examined in this study were from 27 localities (out of 61) spread over various agroecologies (Table 4) in Cameroon. The two identified haplotypes occurred in all agroecologies across Cameroon. Both haplotypes were found together in 48.2% of the surveyed fields, and alone in 37% and 14.8% of the surveyed fields respectively for haplotype 1 (rice strain) and haplotype 2 (corn strain). Corn strain specimens were found in mixed infestation areas (65.4%, n = 26) while rice strain specimens occurred in about the same relative frequency alone (48.9%, n = 45) or in mixed infestation (51.1%, n = 51).

**Table 4:** Occurrence of the Fall armyworm *Spodoptera frugiperda* haplotypes in different agroecological zones in Cameroon.

Agroecology (number of surveyed localities)	Sampled specimens	Haplotype 1 (rice strain)		Haplotype 2 (corn strain)	
		n	%	n	%
Humid forest bimodal rainfall (12)	31	17	54.8	14	45.2
Moist savannah highlands (7)	20	16	80.0	4	20.0
Dry savannah (3)	5	4	80.0	1	20.0
Humid forest monomodal rainfall (5)	15	8	53.3	7	46.7

### Co-occurrence of *S*. *frugiperda* and *Busseola fusca*

**Fig 6:**
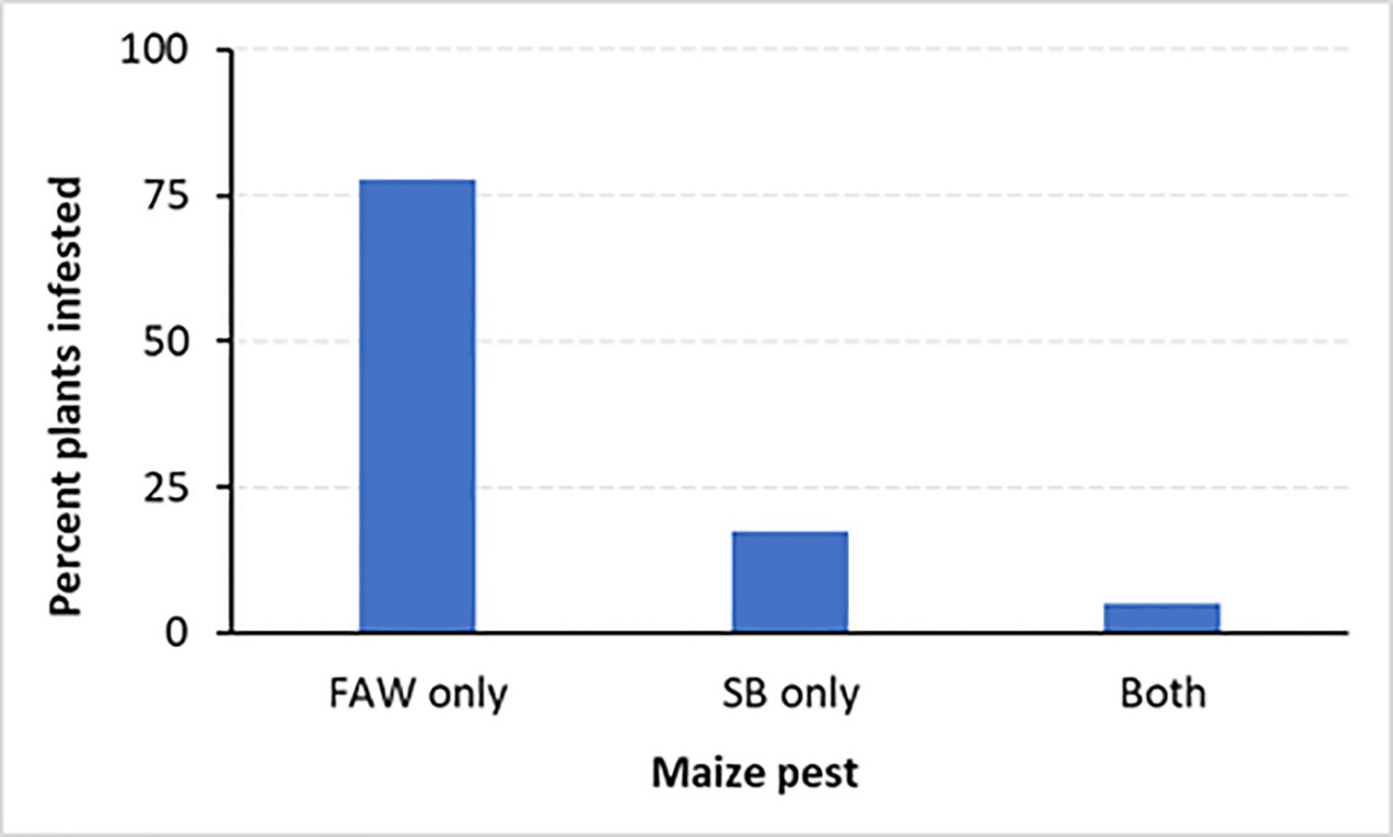
Co-occurrence of *Spodoptera frugiperda* and *Busseola fusca* on maize plants.

*Spodoptera*. *frugiperda* and *B*. *fusca* larvae co-occurred only on 5% of the plants sampled, but in many cases, only *S*. *frugiperda* larvae were recorded on the maize plant (77.6%). *Busseola fusca* occurred alone on 17.4% of the plants (Fig 6).

### Influence of altitude on *S*. *frugiperda* severity

**Fig 7:**
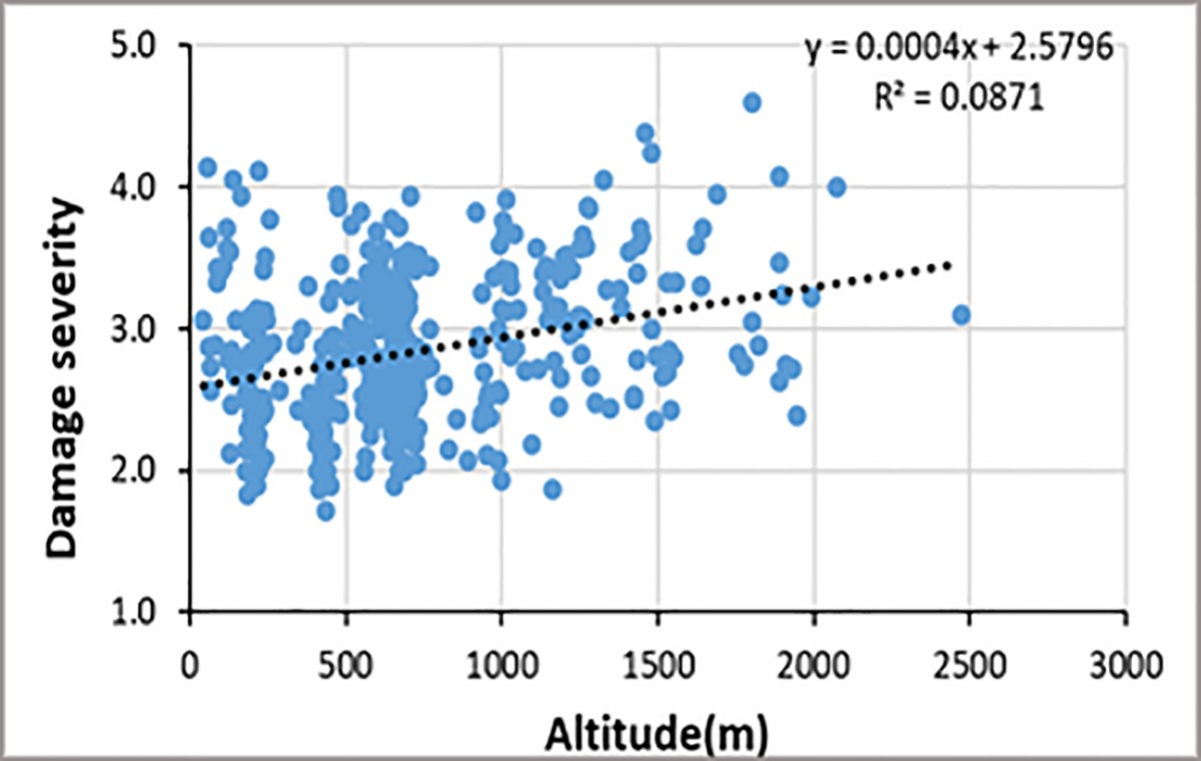
*S*. *frugiperda* severity *vs* altitude in maize fields in Cameroon.

There was no significant association between altitude on *S*. *frugiperda* severity as damage levels were spread nearly equally across all altitudes (Fig 7).

### Host plants

Apart from maize, thirty-one potential host plant species were inspected during the three surveys. *Spodoptera frugiperda* larvae were recorded on six of these 31 plant species: *Sorghum bicolor* (L.) Moench (10.6%; n = 1300), *Solanum tuberosum* L. (2.8%; n = 36), *Ipomoea batatas* (L.) Lam. (1.9%; n = 107), *Saccharum officinarum* L. (0.8%; n = 120), *Phaseolus vulgaris* L. (0.4%; n = 530) and *Gossypium hirsutum* L. (1.9%; n = 1250). (S2 Table)

## Discussion

This study has provided insight in the *S*. *frugiperda* spread, diversity and farmer’s reaction in Cameroon, and possibly similar agroecologies in central Africa since the report of its invasion in 2016. In response to this new threat to maize production, farmers have opted for the application of synthetic pesticides. Although our experiments were not designed to determine pesticide efficacy, as parameters such as time since application were not considered, our observations suggest lack of a drastic effect on *S*. *frugiperda* infestations on maize. Many farmers do not apply the recommended doses or as indicated [37], and in some cases, they use a combination of several insecticides, making it difficult to evaluate their effects. The misuse or overuse of the same pesticide have been cited as major cause of insect resistance to pesticides [38]. In the Americas, resistance to pesticides has been reported for several mode-of-action categories including Carbamates, Organophosphates and Pyrethroids [39,40]. To avoid the occurrence of resistance to pesticides in Africa, farmers should be advised on the proper use of insecticides, includes rates, timing and methods of application. Management of fall armyworm that consider the use of natural enemies and an informed decision of intervention based of moth capture and damage threshold is necessary to avoid resistance that may arise from inappropriate insecticide use [41–43].

There was a variation in incidence and severity among the regions, with a decreasing trend from the humid forest zone towards the Sahelian zone. These observed differences are probably related to haplotype distribution between the regions as shown by the genetics analyses.

Incidence and severity also varied between sampling periods. In bimodal rainfall zones, there seems to be population build up during the first planting season characterized by more growing fields, which also experience severe attack, resulting in greater incidence during the second and the off-season. We could have expected severe attack during the first survey (off-season), due to the reduction maize field size, which are concentrated around swampy areas in association with vegetables, but it was not the case.

At the field level, there was a positive correlation between incidence and severity. Larvae are known to disperse between plants from the egg mass after hatching [44]. Severe damage could result from initial feeding from several larvae or mixed infestation with stemborers. However, at later developmental stages (5^th^ and 6^th^ instar), only one larva was frequently observed per plant and there were few cases of co-occurrence between with stemborers.

Populations of *S*. *frugiperda* Cameroon are grouped into two distinct clades that clustered perfectly with the R (rice) and the M (corn) biotypes provided by [45]. This provide evidence of the co-occurrence of both strains of *S*. *frugiperda* in Cameroon, in contrast to Nigeria, Sao-Tome and Tanzania where only one of the strain was recorded [19,34]. The two strains were recently shown to co-occur also in South Africa [35]. Previous studies in the Americas and in South Africa also showed that both corn and rice types can occur on maize and other crops [35, 46].

While some authors considered the genetic variation between the two *S*. *frugiperda* strains as an intraspecific variation probably due to different introduction events [19], others suggested that the two strains were introduced together over a short period and that the two observed clades represent cryptic species [35]. Although there are considerable data on pheromone composition, different responses in male attraction to pheromones and existence of a premating isolation barrier to support the existence of cryptic species [30,47–49], these differences have been attributed also to geographic differentiation between *S*. *frugiperda* populations and not necessarily strain specific differences [50]. In our DNA barcoding of *S*. *frugiperda* in Cameroon, we found 1.7% sequence divergence between the two *S*. *frugiperda* strains, which is greater than the 1% reported by [34], but it is still below the 2% usually recorded for confirmed congeneric species [51–53]. Intraspecific variation in the South African populations of *S*. *frugiperda* have been also reported, but this variation has not been quantified [35]. While our data is suggestive of the existence of two cryptic species in the *S*. *frugiperda* group in Cameroon, a larger sample is however needed to explore this diversity and to make a definitive conclusion on the existence of cryptic species in Cameroon.

From our samples, we could not make inference on the distribution and abundance of the strains in the agroecologies given the limited number of sample used, especially in the savannah. However uncovering the distribution and abundance of the two strains using larger sample will add knowledge in finding solutions against this new pest of maize in west and central Africa.

*Spodoptera frugiperda* larvae were also recorded on other crops. The species is known to infest over a hundred of plant species, with grasses being the preferred host [24]. This could justify why sorghum was the most infested crop after maize.

While insecticides are likely to remain the first line of defense against *S*. *frugiperda* in Africa, host specific and generalist natural enemies are also potential options to complements the use of selective synthetic chemical by farmers in an integrated approach to the control of S. frugiperda. At present, potential natural enemies like spiders, eggs parasitoids from the family Trichogrammatidae, laval parasitoids from various families (Tachinidae, Phoridae, Braconidae and Ichneumonidae) which were encountered at low density during our surveys do not appear to be effective in controlling *S*. *frugiperda* infestations. Further studies the bioecology of these natural enemies are needed to determine their role in limiting *S*. *frugiperda* populations [39,54–56]. Moreover, there is an urgent need to test available biopesticides and botanical insecticides to supplement available options for the control of *S*. *frugiperda* and reduce losses in the production of maize and other affected crops. Biopesticides and botanicals also provide a low risk alternative to conventional, synthetic pesticides, providing it is supported by a number of rigorously data collection on their efficacy at different maize growth stages and with different *S*. *frugiperda* life stages [57,58]

## Conclusion

*S*. *frugiperda* is present in all regions of Cameroon with two distinct clades that clustered perfectly with the rice and corn strains. Management option should therefore consider the use of more specific natural enemies and an informed decision of intervention based on strain capture and damage threshold, to avoid pesticide resistance that may arise from inadequate use of chemicals. Our observations suggest lack of a drastic effect of pesticide on *S*. *frugiperda* infestations on maize. Current efforts are focusing on identifying potential indigenous natural enemies and screening soft insecticides and biopesticide as part of an integrated control of the pest.

## Supporting information

S1 Table List of Cameroon Fall armyworm and other stemborer specimens collected from maize plants. (PDF)

S2 Table List of FAW host plants other than maize surveyed. (PDF)

S1 Fig *S*. *frugiperda* Incidence *vs* plant age in maize fields in Cameroon. (PDF)

S2 Fig Protein sequence from the COI gene of *S*. *frugiperda* from Cameroon. (PDF)

S3 Fig Phylogenetic relationship of 71 samples of the Fall armyworm *Spodoptera frugiperda* populations from Cameroon. Inferred from the 658 bp mitochondrial cytochrome c oxidase subunit 1 (COI) using Maximum Parsimony method based on the Jukes-Cantor model; The consensus tree inferred from 10 most parsimonious trees is shown. Branches corresponding to partitions reproduced in less than 50% trees are collapsed. The consistency index is 1, the retention index is 1.000000, and the composite index is 1 for all sites and parsimony-informative sites. The percentage of parsimonious trees in which the associated taxa clustered together are shown next to the branches. Evolutionary analyses were conducted in MEGA7.
(PDF)

S4 Fig Phylogenetic relationship of 71 samples of the Fall armyworm *Spodoptera frugiperda* populations from Cameroon. Inferred from the 658 bp mitochondrial cytochrome c oxidase subunit 1 (COI) using Maximum Likelihood method based on the Jukes-Cantor model; The topology of the tree with the highest log likelihood (-1131.5259) is shown). The tree is drawn to scale, with branch lengths measured in the number of substitutions per site. The percentage of replicate trees in which the associated taxa clustered together in the bootstrap test (1000 replicates) is shown next to the branches. Initial tree(s) for the heuristic search were obtained automatically by applying the Maximum Parsimony method. Evolutionary analyses were conducted in MEGA7.
(PDF)

S5 Fig Phylogenetic relationship of 71 samples of the Fall armyworm *Spodoptera frugiperda* and 24 samples of other stemborer populations from Cameroon. Inferred from the 658 bp mitochondrial cytochrome c oxidase subunit 1 (COI) using Neighbor-Joining method based on p-distances; The optimal tree with the sum of branch length = 0.36286936 is shown. The percentage of replicate trees in which the associated taxa clustered together in the bootstrap test (1000 replicates) are shown next to the branches. Scale unit is in the number of base differences per site. Evolutionary analyses were conducted in MEGA7.
(PDF)
