# Correction: *Spodoptera frugiperda* Smith (Lepidoptera: Noctuidae) in Cameroon: Case study on its distribution, damage, pesticide use, genetic differentiation and host plants

**DOI:** 10.1371/journal.pone.0217653

**Published:** 2019-06-04

**Authors:** Apollin Fotso Kuate, Rachid Hanna, Armand R. P. Doumtsop Fotio, Albert Fomumbod Abang, Samuel Nanga Nanga, Sergine Ngatat, Maurice Tindo, Cargele Masso, Rose Ndemah, Christopher Suh, Komi Kouma Mokpokpo Fiaboe

The images for Figs 3 and 4 are incorrectly switched. The image that appears as Fig 3 should be Fig 4, and the image that appears as Fig 4 should be Fig 3. The figure captions appear in the correct order.

**Fig 3:**
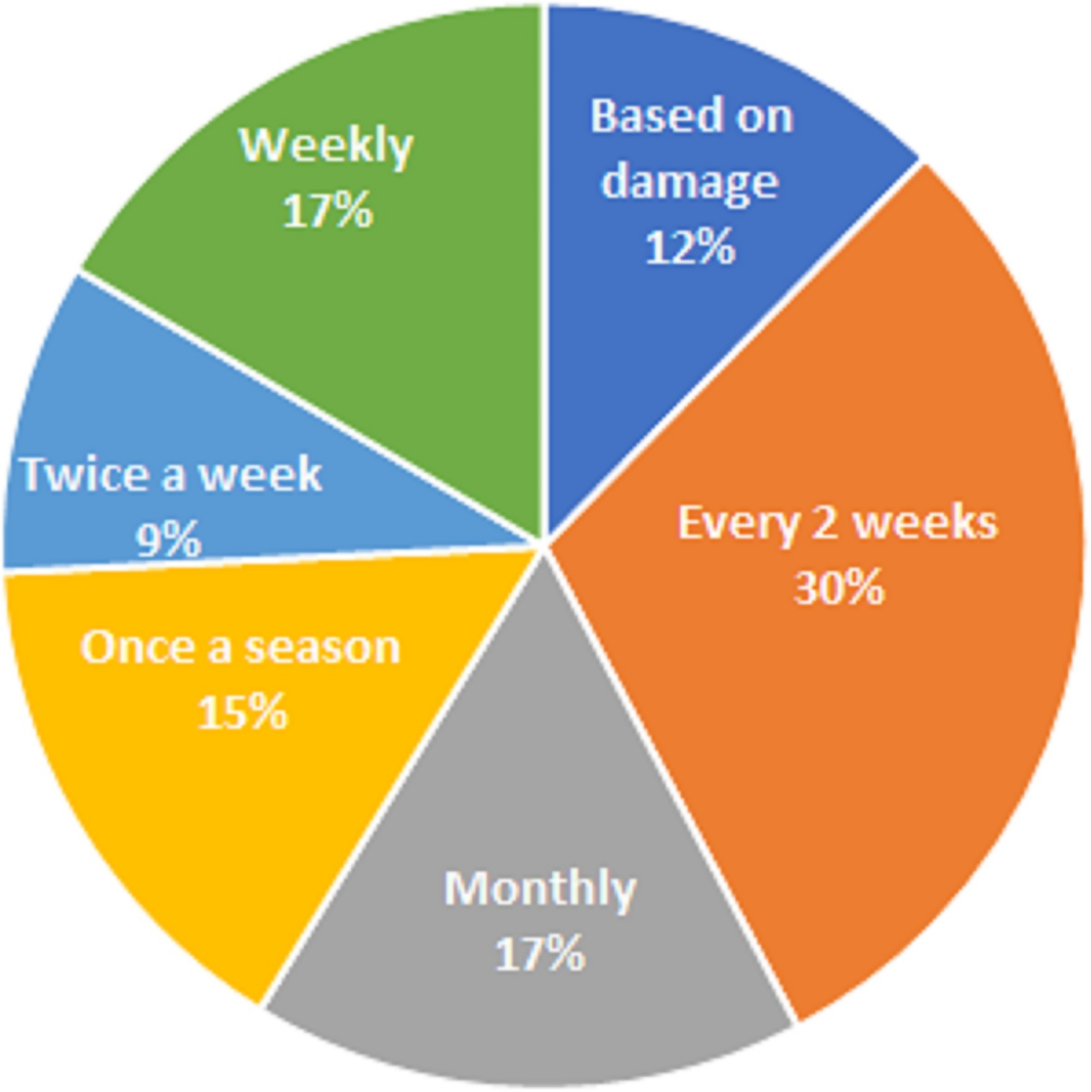
Pesticide application frequency to control *S*. *frugiperda* on maize by farmers.

**Fig 4:**
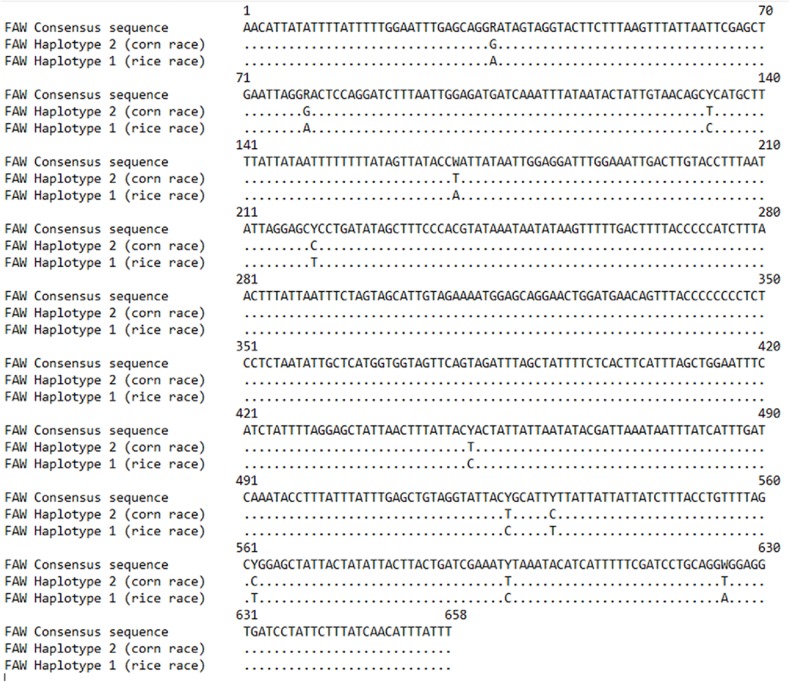
Identity plot of the COI sequences of *S*. *frugiperda* haplotypes from Cameroon.
