# Supplementary material for: Spodoptera frugiperda Smith (Lepidoptera: Noctuidae) in Cameroon: Case study on its distribution, damage, pesticide use, genetic differentiation and host plants
Source: PLoS One. 2019 Apr 29;14(4):e0215749. doi: 10.1371/journal.pone.0215749 (PMC6488053; doi:10.1371/journal.pone.0215749)
Supplement: S1 Table — (PDF) [file pone.0215749.s001.pdf]

S1 Table. List of Cameroon Fall armyworm and other stemborer specimens collected from maize plants.

No	Accession code	*Species*	Locality	Identity %	FAW Race
1	S1 FAW_Zoetele	*S. frugiperda*	Zoetele	100	Rice race
2	S2 SB_Zoetele	*Sesamia calamistis*	Zoetele	98	
3	S3 SB_Ayos	*Helicoverpa armigera*	Ayos	100	
4	S4 SB_Ayos	*Sesamia calamistis*	Ayos	98	
5	S5 FAW_Ekona	*S. frugiperda*	Ekona	100	Rice race
6	S6 SB_Ekona	*Sesamia calamistis*	Ekona	98	
7	S7 FAW_Kumbo	*S. frugiperda*	Kumbo	100	Corn race
8	S8 SB_Kumbo	*Busseola fusca*	Kumbo	100	
9	S9 SB_Ambam	*Busseola fusca*	Ambam	100	
10	S10 FAW_Ambam	*S. frugiperda*	Ambam	100	Corn race
11	S11 SB_Garoua Boulai	*Busseola fusca*	Garoua Boulai	99	
12	S12 SB_Meiganga	*Busseola fusca*	Meiganga	100	
13	S13 FAW_Meiganga	*S. frugiperda*	Meiganga	100	Rice race
14	S14 SB_Bertoua	*Sesamia calamistis*	Bertoua	98	
15	S15 FAW_Bertoua	*S. frugiperda*	Bertoua	100	Rice race
16	S16 FAW_Dschang	*S. frugiperda*	Dschang	100	Corn race
17	S17 SB_Dschang	*Busseola fusca*	Dschang	100	
18	S18 SB_Bambui	*Busseola fusca*	Bambui	99	
19	S19 FAW_Bambui	*S. frugiperda*	Bambui	100	Rice race
20	S20 SB_Kumbo	*Busseola fusca*	Kumbo	100	
21	S21 FAW_Kumbo	*S. frugiperda*	Kumbo	100	Corn race
22	S22 FAW_Ndop	*S. frugiperda*	Ndop	100	Rice race
23	S23 SB_Ndop	*Busseola fusca*	Ndop	100	
24	S24 FAW_Abang Minko'o	*S. frugiperda*	Abang Minko'o	100	Rice race
25	S25 FAW_Abang Minko'o	*S. frugiperda*	Abang Minko'o	100	Corn race
26	S26 FAW_Nkoevone	*S. frugiperda*	Nkoevone	100	Rice race
27	S27 FAW_Nkoevone	*S. frugiperda*	Nkoevone	100	Corn race
28	S28 FAW_Sangmelima	*S. frugiperda*	Sangmelima	100	Corn race
29	S29 FAW_Sangmelima	*S. frugiperda*	Sangmelima	100	Corn race
30	S30 FAW_Ebolowa	*S. frugiperda*	Ebolowa	100	Rice race
31	S31 FAW_Ebolowa	*S. frugiperda*	Ebolowa	100	Rice race
32	S32 FAW_Zoetele	*S. frugiperda*	Zoetele	100	Corn race
33	S33 FAW_Ntui	*S. frugiperda*	Ntui	100	Corn race

No	Accession code	*Species*	Locality	Identity %	FAW Race
34	S34 FAW_Ntui	*S. frugiperda*	Ntui	100	Rice race
35	S35 FAW_Ayos	*S. frugiperda*	Ayos	100	Corn race
36	S36 FAW_Bertoua	*S. frugiperda*	Bertoua	100	Rice race
37	S37 FAW_Bertoua	*S. frugiperda*	Bertoua	100	Rice race
38	S38 FAW_Garoua Boulai	*S. frugiperda*	Garoua Boulai	100	Rice race
39	S39 SB_Garoua Boulai	*Busseola fusca*	Garoua Boulai	100	
40	S40 FAW_|Meiganga	*S. frugiperda*	Meiganga	100	Rice race
41	S41 FAW_Benoue	*S. frugiperda*	Benoue	100	Rice race
42	S42 SB_Benoue	*Sesamia cretica*	Benoue	100	
43	S43 FAW_Nkolbisson	*S. frugiperda*	Nkolbisson	100	Rice race
44	S44 SB_Njombe	*Eldana saccharina*	Njombe	99	
45	S45 FAW_Ekona	*S. frugiperda*	Ekona	100	Corn race
46	S46 FAW_Kumba	*S. frugiperda*	Kumba	100	Corn race
47	S47 SB_Kumba	*Sesamia calamistis*	Kumba	98	
48	S48 FAW_Mamfe	*S. frugiperda*	Mamfe	100	Rice race
49	S49 FAW_Bambui	*S. frugiperda*	Bambui	100	Rice race
50	S50 SB_Bambui	*Sesamia calamistis*	Bambui	98	
51	S51 FAW_Ndop	*S. frugiperda*	Ndop	100	Corn race
52	S52 FAW_Ndop	*S. frugiperda*	Ndop	100	Rice race
54	S54 FAW_Foumbot	*S. frugiperda*	Foumbot	100	Rice race
55	S55 FAW_Sanchou	*S. frugiperda*	Sanchou	100	Rice race
56	S56 SB_Sanchou	*Busseola fusca*	Sanchou	99	
57	S57 FAW_Dschang	*S. frugiperda*	Dschang	100	Rice race
58	S58 FAW_Dschang	*S. frugiperda*	Dschang	100	Rice race
59	S59 FAW_Bafoussam	*S. frugiperda*	Bafoussam	100	Rice race
60	S60 SB_Ambam	*Busseola fusca*	Ambam	100	
61	S61 FAW_Nkoevone	*S. frugiperda*	Nkoevone	100	Rice race
62	S62 FAW_Nkoevone	*S. frugiperda*	Nkoevone	100	Rice race
63	S63 SB_Ebolowa	*Busseola fusca*	Ebolowa	100	
64	S64 FAW_Ebolowa	*S. frugiperda*	Ebolowa	100	Rice race
65	S65 FAW_Mbalmayo	*S. frugiperda*	Mbalmayo	100	Corn race
66	S66 FAW_Mbalmayo	*S. frugiperda*	Mbalmayo	100	Rice race
67	S67 FAW_Garoua Boulai	*S. frugiperda*	Garoua Boulai	100	Corn race
68	S68 FAW_Ayos	*S. frugiperda*	Ayos	100	Corn race
69	S69 FAW_Sangmelima	*S. frugiperda*	Sangmelima	100	Corn race
70	S70 FAW_Sangmelima	*S. frugiperda*	Sangmelima	100	Corn race
71	S71 FAW_Zoetele	*S. frugiperda*	Zoetele	100	Corn race
72	S72 FAW_Zoetele	*S. frugiperda*	Zoetele	100	Rice race
73	S73 FAW_Ntui	*S. frugiperda*	Ntui	100	Corn race

No	Accession code	*Species*	Locality	Identity %	FAW Race
74	S74 FAW_Foumbot	*S. frugiperda*	Foumbot	100	Rice race
75	S75 FAW_Foumbot	*S. frugiperda*	Foumbot	100	Rice race
76	S76 FAW_Bafoussam	*S. frugiperda*	Bafoussam	100	Rice race
77	S77 FAW_Bafoussam	*S. frugiperda*	Bafoussam	100	Rice race
78	S78 SB_Bafoussam	*Busseola fusca*	Bafoussam	99	
79	S79 SB_Dschang	*Busseola fusca*	Dschang	100	
80	S80 FAW_Sanchou	*S. frugiperda*	Sanchou	100	Corn race
81	S81 FAW_Sanchou	*S. frugiperda*	Sanchou	100	Rice race
82	S82 FAW-Njombe	*S. frugiperda*	Njombe	100	Corn race
83	S83 FAW_Njombe	*S. frugiperda*	Njombe	100	Rice race
84	S84 FAW_Ekona	*S. frugiperda*	Ekona	100	Corn race
85	S85 FAW_Ekona	*S. frugiperda*	Ekona	100	Rice race
86	S86 FAW_Kumba	*S. frugiperda*	Kumba	100	Rice race
87	S87 FAW_Kumba	*S. frugiperda*	Kumba	100	Corn race
88	S88 FAW_Mamfe	*S. frugiperda*	Mamfe	100	Rice race
89	S89 FAW_Mamfe	*S. frugiperda*	Mamfe	100	Corn race
90	S90 FAW_Bambui	*S. frugiperda*	Bambui	100	Rice race
91	S91 FAW_Ndop	*S. frugiperda*	Ndop	100	Rice race
92	S92 SB_Mbalmayo	*S. littoralis*	Mbalmayo	100	
93	S93 FAW_Mbalmayo	*S. frugiperda*	Mbalmayo	100	Rice race
94	S94 FAW_Meyomessala	*S. frugiperda*	Meyomessala	100	Rice race
95	S95 FAW_Bafoussam	*S. frugiperda*	Bafoussam	100	Rice race
96	S96 FAW_Zilin	*S. frugiperda*	Zilin	100	Rice race
