# Supplementary material for: Spodoptera frugiperda Smith (Lepidoptera: Noctuidae) in Cameroon: Case study on its distribution, damage, pesticide use, genetic differentiation and host plants
Source: PLoS One. 2019 Apr 29;14(4):e0215749. doi: 10.1371/journal.pone.0215749 (PMC6488053; doi:10.1371/journal.pone.0215749)
Supplement: S2 Table — (PDF) [file pone.0215749.s002.pdf]

**S2 Table. List of FAW host plants other than maize surveyed**

| Host plants surveyed | # plants | FAW occurrence | % |
|---|---|---|---|
| *Sorghum bicolor* (L.) Moench | 1300 | 139 | 10.7 |
| *Solanum tuberosum* L. | 36 | 1 | 2.8 |
| *Gossypium hirsutum* L. | 1250 | 24 | 1.9 |
| *Ipomoea batatas* (L.) Lam. | 107 | 2 | 1.9 |
| *Saccharum officinarum* L | 120 | 1 | 0.8 |
| *Phaseolus vulgaris* L. | 530 | 2 | 0.4 |
| Amaranthus | 109 | 0 | 0 |
| *Andropogon gayanus* | 60 | 0 | 0 |
| Cabbage | 12 | 0 | 0 |
| *Mariscus flabeliformis* | 12 | 0 | 0 |
| *Cetaria barbata* | 132 | 0 | 0 |
| Cowpea | 24 | 0 | 0 |
| *Cyperus diformis* | 12 | 0 | 0 |
| *Solanum macrocarpon* | 36 | 0 | 0 |
| *Pennisetum purpureum* | 252 | 0 | 0 |
| *Pannicum maximum* | 24 | 0 | 0 |
| *Arachis hypogea* | 288 | 0 | 0 |
| *Mucuna pruriens* | 84 | 0 | 0 |
| *Musa parasidiaca* | 24 | 0 | 0 |
| *Penicetum violaceum* | 96 | 0 | 0 |
| *Penicetum pedicellarum* | 396 | 0 | 0 |
| *Capsicum frutescens* | 84 | 0 | 0 |
| *Rottboellia cochinchinensis* | 24 | 0 | 0 |
| *Solanum nigrum* | 72 | 0 | 0 |
| Soybean | 12 | 0 | 0 |
| *Cucumis melo (melon)* | 24 | 0 | 0 |
| Solanum sp (nightshade) | 24 | 0 | 0 |
| *Cymbopogon citratus* | 12 | 0 | 0 |
| Tomato | 48 | 0 | 0 |
| Water melon | 36 | 0 | 0 |
