# Supplementary figures and images for: Spodoptera frugiperda Smith (Lepidoptera: Noctuidae) in Cameroon: Case study on its distribution, damage, pesticide use, genetic differentiation and host plants
Source: PLoS One. 2019 Apr 29;14(4):e0215749. doi: 10.1371/journal.pone.0215749 (PMC6488053; doi:10.1371/journal.pone.0215749)

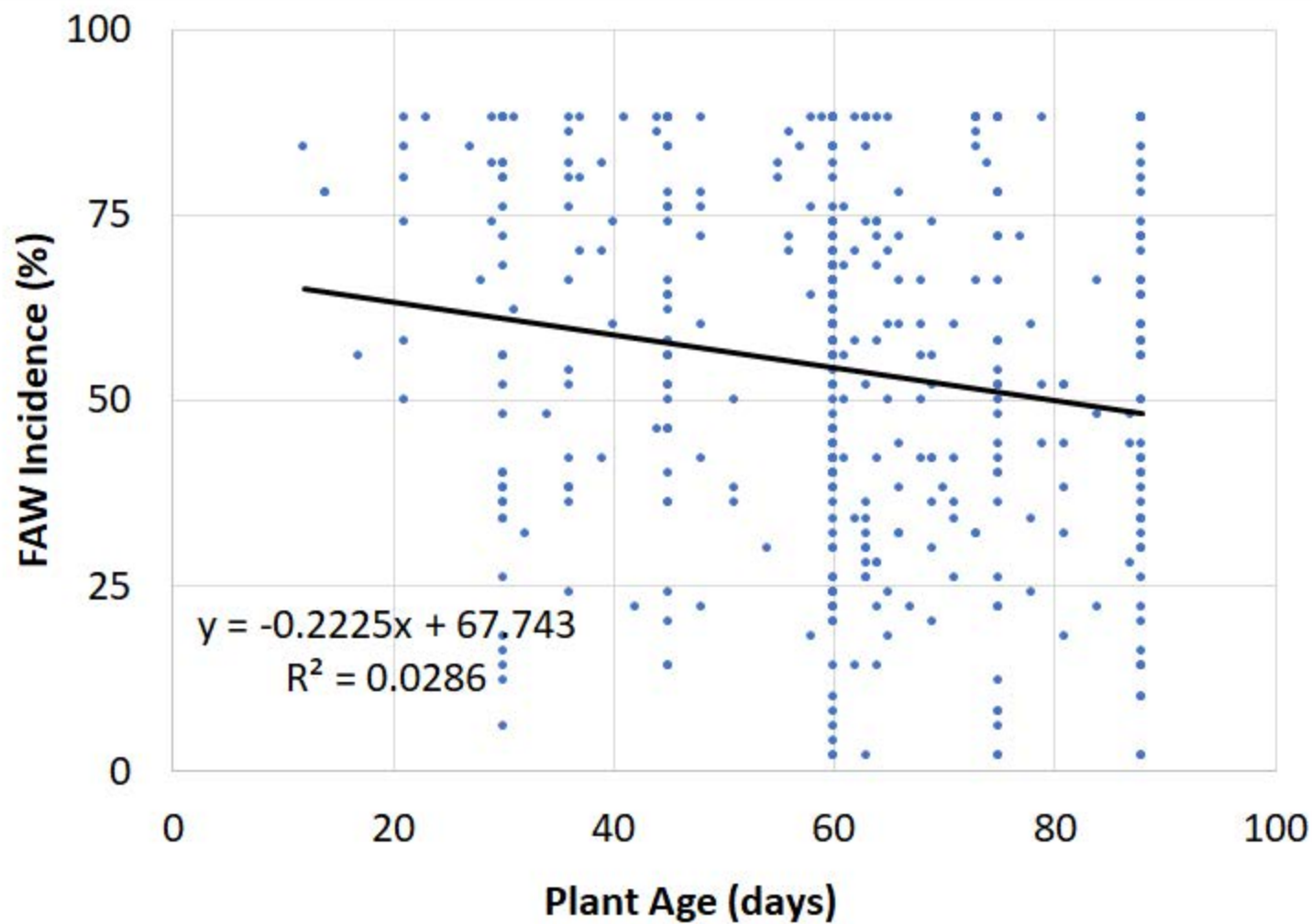

Supplement: S1 Fig — (PDF) [file pone.0215749.s003.pdf]
