# Supplementary material for: Spodoptera frugiperda Smith (Lepidoptera: Noctuidae) in Cameroon: Case study on its distribution, damage, pesticide use, genetic differentiation and host plants
Source: PLoS One. 2019 Apr 29;14(4):e0215749. doi: 10.1371/journal.pone.0215749 (PMC6488053; doi:10.1371/journal.pone.0215749)
Supplement: S2 Fig — (PDF) [file pone.0215749.s004.pdf]

S2 Fig. Protein sequence from the COI gene of *S. frugiperda* from Cameroon.

```
                     1                                                                    70
FAW COI protein      TLYFIFGIWAGMVGTSLSLLIRAELGTPGSLIGDDQIYNTIVTAHAFIMIFFMVMPIMIGGFGNWLVPLM
                    71                                                                   140
FAW COI protein      LGAPDMAFPRMNNMSFWLLPPSLTLLISSSIVENGAGTGWTVYPPLSSNIAHGGSSVDLAIFSLHLAGIS
                   141                                                                   210
FAW COI protein      SILGAINFITTIINMRLNNLSFDQMPLFIWAVGITAFLLLLSLPVLAGAITMLLTDRNLNTSFFDPAGGG
                   211       219
FAW COI protein      DPILYQHLF
```
