# Supplementary material for: Spodoptera frugiperda Smith (Lepidoptera: Noctuidae) in Cameroon: Case study on its distribution, damage, pesticide use, genetic differentiation and host plants
Source: PLoS One. 2019 Apr 29;14(4):e0215749. doi: 10.1371/journal.pone.0215749 (PMC6488053; doi:10.1371/journal.pone.0215749)

S3 Fig. Phylogenetic relationship of 71 samples of the Fall armyworm *Spodoptera frugiperda* populations from Cameroon. Inferred from the 658 bp mitochondrial cytochrome c oxidase subunit 1 (COI) using Maximum Parsimony method based on the Jukes-Cantor model; The consensus tree inferred from 10 most parsimonious trees is shown. Branches corresponding to partitions reproduced in less than 50% trees are collapsed. The consistency index is 1, the retention index is 1.000000, and the composite index is 1 for all sites and parsimony-informative sites. The percentage of parsimonious trees in which the associated taxa clustered together are shown next to the branches. Evolutionary analyses were conducted in MEGA7.

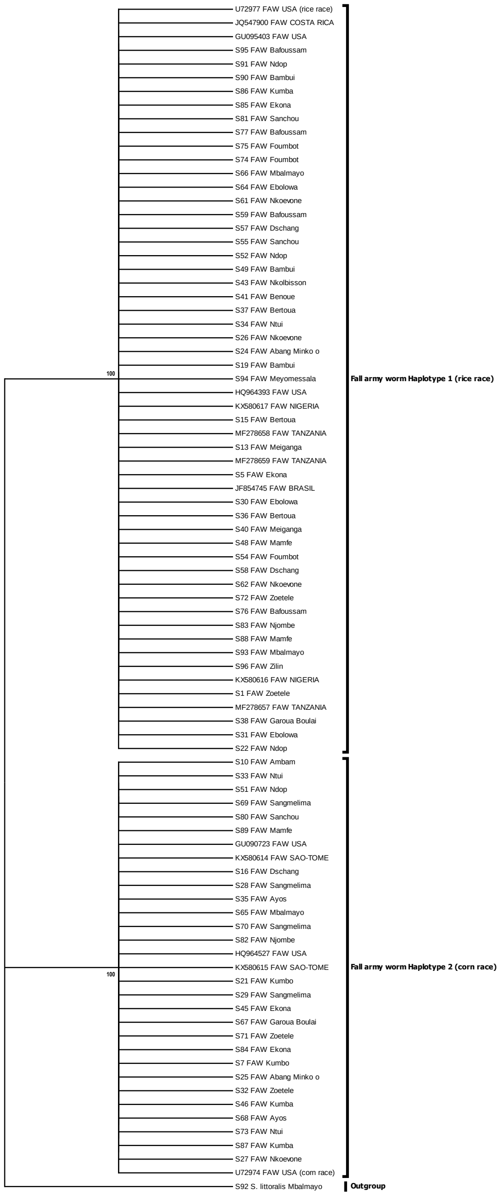

Supplement: S3 Fig — Inferred from the 658 bp mitochondrial cytochrome c oxidase subunit 1 (COI) using Maximum Parsimony method based on the Jukes-Cantor model; The consensus tree inferred from 10 most parsimonious trees is shown. Branches corresponding to partitions reproduced in less than 50% trees are collapsed. The consistency index is 1, the retention index is 1.000000, and the composite index is 1 for all sites and parsimony-informative sites. The percentage of parsimonious trees in which the associated taxa clustered together are shown next to the branches. Evolutionary analyses were conducted in MEGA7. (PDF) [file pone.0215749.s005.pdf]
