# Supplementary material for: Spodoptera frugiperda Smith (Lepidoptera: Noctuidae) in Cameroon: Case study on its distribution, damage, pesticide use, genetic differentiation and host plants
Source: PLoS One. 2019 Apr 29;14(4):e0215749. doi: 10.1371/journal.pone.0215749 (PMC6488053; doi:10.1371/journal.pone.0215749)

S4 Fig. Phylogenetic relationship of 71 samples of the Fall armyworm *Spodoptera frugiperda* populations from Cameroon. Inferred from the 658 bp mitochondrial cytochrome c oxidase subunit 1 (COI) using Maximum Likelihood method based on the Jukes-Cantor model; The topology of the tree with the highest log likelihood (-1131.5259) is shown). The tree is drawn to scale, with branch lengths measured in the number of substitutions per site. The percentage of replicate trees in which the associated taxa clustered together in the bootstrap test (1000 replicates) is shown next to the branches. Initial tree(s) for the heuristic search were obtained automatically by applying the Maximum Parsimony method. Evolutionary analyses were conducted in MEGA7

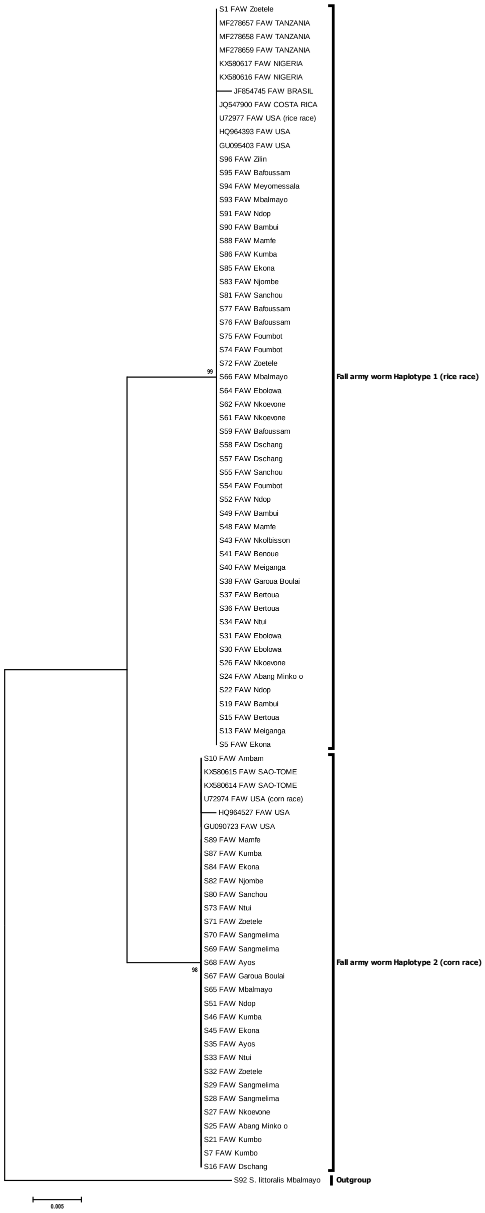

Supplement: S4 Fig — Inferred from the 658 bp mitochondrial cytochrome c oxidase subunit 1 (COI) using Maximum Likelihood method based on the Jukes-Cantor model; The topology of the tree with the highest log likelihood (-1131.5259) is shown). The tree is drawn to scale, with branch lengths measured in the number of substitutions per site. The percentage of replicate trees in which the associated taxa clustered together in the bootstrap test (1000 replicates) is shown next to the branches. Initial tree(s) for the heuristic search were obtained automatically by applying the Maximum Parsimony method. Evolutionary analyses were conducted in MEGA7. (PDF) [file pone.0215749.s006.pdf]
