# Supplementary material for: Spodoptera frugiperda Smith (Lepidoptera: Noctuidae) in Cameroon: Case study on its distribution, damage, pesticide use, genetic differentiation and host plants
Source: PLoS One. 2019 Apr 29;14(4):e0215749. doi: 10.1371/journal.pone.0215749 (PMC6488053; doi:10.1371/journal.pone.0215749)

S5 Fig. Phylogenetic relationship of 71 samples of the Fall armyworm *Spodoptera frugiperda* and 24 samples of other stemborer populations from Cameroon. Inferred from the 658 bp mitochondrial cytochrome c oxidase subunit 1 (COI) using Neighbor-Joining method based on p-distances; The optimal tree with the sum of branch length = 0.36286936 is shown. The percentage of replicate trees in which the associated taxa clustered together in the bootstrap test (1000 replicates) are shown next to the branches. Scale unit is in the number of base differences per site. Evolutionary analyses were conducted in MEGA7.

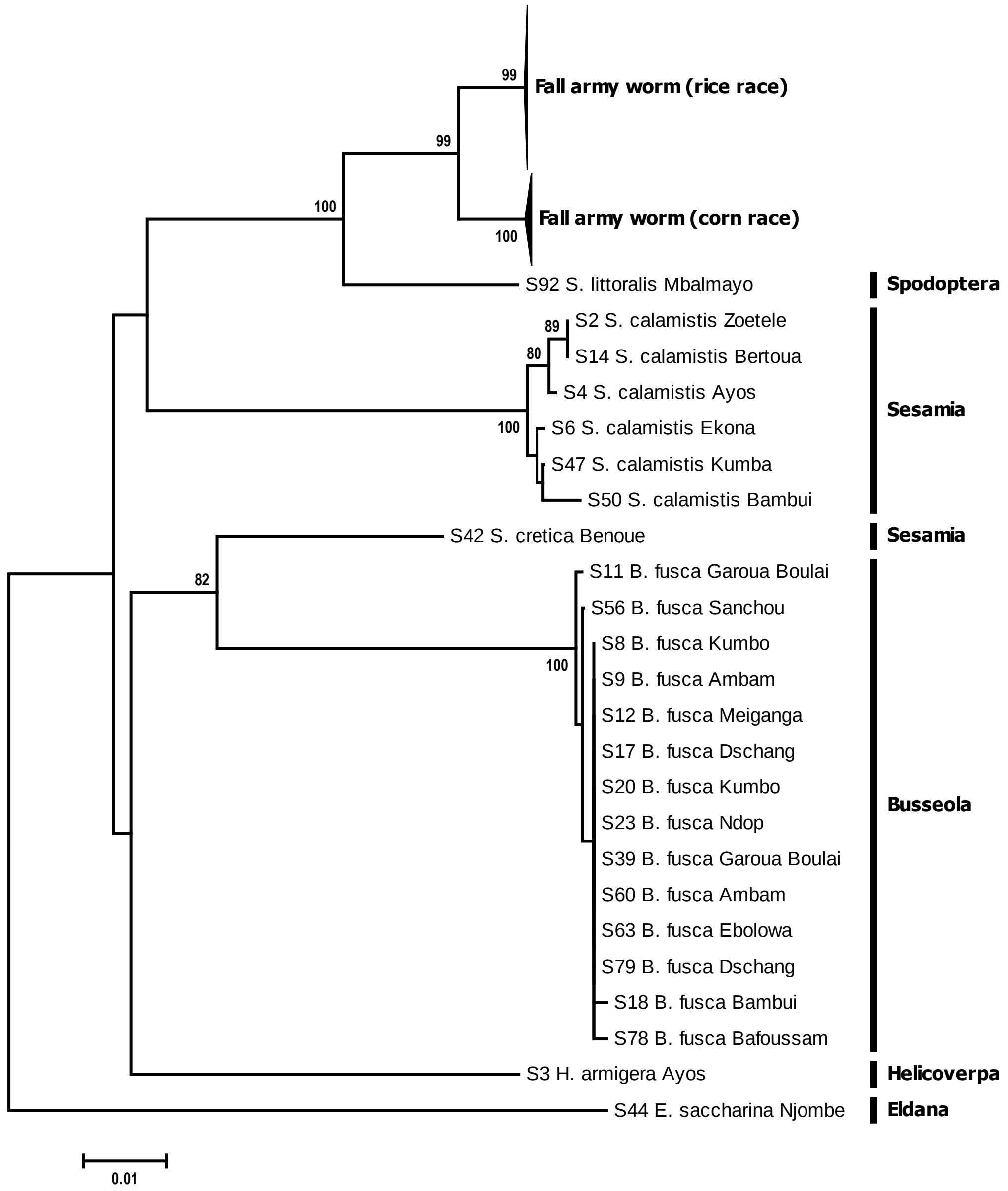

Supplement: S5 Fig — Inferred from the 658 bp mitochondrial cytochrome c oxidase subunit 1 (COI) using Neighbor-Joining method based on p-distances; The optimal tree with the sum of branch length = 0.36286936 is shown. The percentage of replicate trees in which the associated taxa clustered together in the bootstrap test (1000 replicates) are shown next to the branches. Scale unit is in the number of base differences per site. Evolutionary analyses were conducted in MEGA7. (PDF) [file pone.0215749.s007.pdf]
